# Changes in soil microbial communities after exposure to neonicotinoids: A systematic review

**DOI:** 10.1111/1758-2229.13193

**Published:** 2023-08-13

**Authors:** Sharmin Akter, Nilantha R. Hulugalle, Julia Jasonsmith, Craig L. Strong

**Affiliations:** ^1^ Fenner School of Environment and Society, College of Science Australian National University Canberra ACT Australia; ^2^ Soil Resource Development Institute Ministry of Agriculture Dhaka Bangladesh

## Abstract

Neonicotinoids are a group of nicotine‐related chemicals widely used as insecticides in agriculture. Several studies have shown measurable quantities of neonicotinoids in the environment but little is known regarding their impact on soil microbial populations. The purpose of this systematic review was to clarify the effects of neonicotinoids on soil microbiology and to highlight any knowledge gaps. A formal systematic review was performed following PRISMA (Preferred Reporting Items for Systematic Review and Meta‐Analyses) guidelines using keywords in PubMed, SCOPUS and Web of Science. This resulted in 29 peer‐reviewed articles, whose findings diverged widely because of variable methodologies. Field‐based studies were few (28%). Imidacloprid was the most widely used (66%) and soil microbial communities were most sensitive to it. Spray formulations were used in 83% of the studies and seed treatments in the rest. Diversity indices were the most frequently reported soil microbial parameter (62%). About 45% of the studies found that neonicotinoids had adverse impacts on soil microbial community structure, composition, diversity, functioning, enzymatic activity and nitrogen transformation. Interactions with soil physicochemical properties were poorly addressed in all studies. The need for more research, particularly field‐based research on the effects of neonicotinoids on soil microorganisms was highlighted by this review.

## Graphical abstract

This systematic review aimed to elucidate neonicotinoid impact on soil microbiology and identify knowledge gaps. Only 29 peer‐reviewed articles were identified as suitable for inclusion in this study, of which 45% reported that neonicotinoids had negative impacts on soil microbial community composition, structure, diversity, functioning, enzymatic activity and nitrogen transformation. The paucity of published studies highlighted the need for more research on this particular topic.

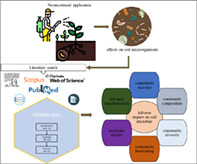

## INTRODUCTION

In agriculture, crop quality and yield depend to a large extent on crop protection. Crop protection is defined as management practices that protect crops from weeds, pests and pathogens. Annually about 26%–40% of global potential crop yield may be lost due to weeds, pests and diseases (OECD/FAO, 2012) and therefore the losses could certainly be twofold without crop protection. Thus, to secure good quality and quantity crop production, farmers use pesticides from both natural and synthetic sources. Neonicotinoids are nicotine‐moiety‐based systemic insecticides (Kasiotis & Machera, 2015) that have been widely used globally since the early 1990s (Goulson, 2013). Structurally, neonicotinoids are hydroheterocyclic guanidines/ amidines conjugated with an aromatic heterocyclic group, electron‐withdrawing group and elastic bond (Buszewski et al., 2019). They function as nicotinic acetylcholine receptor agonists by targeting the sodium/potassium ionophore of an insect's central nervous system and disrupting cholinergic neurotransmission (Jeschke et al., 2011; Moffat et al., 2016; Seifert, 2014). Neonicotinoids are popular over other insecticides because of their ease of application, toxicity to a wide range of insects, high potency for controlling insects and low acute toxicity to mammalians (Thompson et al., 2020). Based on the primary site of action, there are seven active ingredients of neonicotinoids commercially available in the global pesticide market viz. dinotefuran, clothianidin, thiacloprid, thiamethoxam, acetamiprid, nitenpyram and imidacloprid (IRAC, 2022).

Although neonicotinoids are intended to be taken up by the plant, a greater portion of the applied insecticide failed to reach the target plant tissue (Sur & Stork, 2003). Only about 5% of the applied active ingredients of neonicotinoids are taken up by the target plant and remaining majority dispersing to the surrounding environment in soil, air and water (Wood & Goulson, 2017). Neonicotinoids have a limited propensity for volatilization, which means during spray treatments, they are most likely to exist in a gaseous form for a short period of time (Bonmatin et al., 2015). These compounds are transported in the soil environment primarily by surface runoff, leaching and plant uptake (Borsuah et al., 2020; Pietrzak et al., 2020). It had been reported by Chrétien et al. (2017) that surface runoff was a significant transportation mode that contributed about 53% of the observed total losses of neonicotinoids. After entering the soil, neonicotinoids may undergo a number of processes: leaching through the soil profile, sorption, dissipation and degradation (Anderson et al., 2015; Dankyi et al., 2018; Pietrzak et al., 2020). The leaching behaviour of neonicotinoids is predominantly determined by sorption and desorption processes. Sorption affinities are generally influenced by soil organic carbon, soil texture and soil temperature (Anderson et al., 2015; Zhang et al., 2018). Degradation is also a crucial factor in determining how pesticides behave in soils. Neonicotinoids can be degraded abiotically viz. physical, photochemical and chemical degradation, biodegradation (plants and microorganisms), or through enzymatic activity by producing different metabolites (Zhang et al., 2018). Due to low volatility and high water solubility nature, neonicotinoids may leach into the surface and groundwater, raising safety concerns (Bonmatin et al., 2015).

To gain approval for use in agricultural systems, pesticides should be used in such a way that their residues do not accumulate in the surrounding environment with a minimal impact on non‐target organisms (Lo, 2010). Many queries have been raised in recent decades concerning the possible effect of neonicotinoids on non‐target species. Some studies revealed that neonicotinoids had some detrimental impacts on agriculturally useful species, such as earthworms and insect pollinators, notably honeybees (James et al., 2016; Pisa et al., 2015). A pesticide's fate in soil depends on physicochemical properties like solubility, adsorption, persistence, volatility, etc. The physicochemical properties of neonicotinoids like octanol/water partition coefficient (log *K*_ow_) and dissociation constant (pKa) enable them to be systemic in nature, and hence, facilitate their pathway into plant tissues and their movement to all parts of plants (Simon‐delso et al., 2015). A wide range of log *K*_ow_ values (−0.55 to 1.26) and long half‐lives (DT_50_ value 3–545 days) also makes neonicotinoids potential to transport offsite (Hladik & Kolpin, 2016). So, being highly soluble in water, neonicotinoids can easily move downward and persist in the soil from days to years depending on formulations, soil texture, organic matter content, management practices and climatic conditions (Bonmatin et al., 2015). The downward movement usually occurs from the topsoil, which is considered as the region of greater microbial activity and the residues of neonicotinoids may pose a threat to soil microorganisms.

The term ‘soil microbes’ or ‘soil microorganisms’ refers to the diverse community of microorganisms and is broadly defined as a group of microscopic life forms that include bacteria, archaea, viruses and eukaryotes such as fungi (Flombaum et al., 2022). In agriculture, soil microorganisms are important in biogeochemical cycling, hence alteration in microbial populations eventually affects soil fertility and crop production. Microbial communities are analysed using a variety of methods, of which, microbial activity and abundance are well‐known indices of soil quality (Lori et al., 2017). Neonicotinoids may potentially affect soil microbial populations in terms of diversity and abundance (Yu et al., 2020) and biochemical functioning (Cycoń & Piotrowska‐Seget, 2015b; Mahapatra et al., 2017).

Individual study outcomes are likely to change due to experimental characteristics such as neonicotinoid active ingredients, treatment rates, soil type, or even the environmental circumstances in which the experiments are conducted (Bonmatin et al., 2015). Research on the effects of neonicotinoids on soil microbes is progressing, but a systematic assessment of neonicotinoid impacts on microbial activities and population in soils has not been previously reported. This systematic review aims to provide an understanding of neonicotinoids' impacts on soil microbiology and thus identify the disparities in the body of knowledge. The objective of this multi‐approach systematic review was to analyse all available publications that investigated the changes in soil microbial communities after applying neonicotinoid insecticides.

## METHODS

### Literature exploration and appraisal

#### Literature searching strategy

We accessed the available literatures on the impacts of neonicotinoid insecticides on soil microbial communities included in SCOPUS, Web of Science and PubMed databases following PRISMA guidelines^1^ (Page et al., 2021). The PRISMA flow diagram was prepared by accessing an online tool (https://estech.shinyapps.io/prisma_flowdiagram/). The literature search was conducted by using the keywords ‘neonicotinoids AND soil AND microorganisms’, ‘imidacloprid OR clothianidin OR thiamethoxam OR acetamiprid OR thiacloprid OR dinotefuran OR nitenpyram AND soil AND microbial AND communities’, ‘neonicotinoids AND soil AND bacterial AND communities’ and ‘neonicotinoids AND soil AND fungal AND communities’ restricted to the publication date between 2000 and 2022. The search results were collated and a reference list was made for screening the titles and abstracts.

#### Screening of studies

Preliminarily identified articles were screened by reading the full texts for further assessment. Only papers that met the following criteria were included in this review:

1. The research must be a published original research paper in a peer‐reviewed journal and available in English;
2. Applied neonicotinoid to the soil as formulation or seed treatment;
3. Provided soil microbial community‐related data;
4. Applied appropriate controls as well as replication.

### Extraction of data

Articles were selected based on the above‐mentioned eligibility criteria. For each study, the following data were collected: title of the study, name of the authors, journal name, year of publication, type of neonicotinoids with exposure modality, study design, physicochemical properties, microbiological parameters and geographical location of sampling sites. Soil physicochemical properties included soil pH (in water), organic carbon (g/100 g), soil texture along with clay, silt and sand concentrations (g/100 g), and soil depth (m). The following formula by Lierop (1981) was used to convert pH values in CaCl_2_ to pH in H_2_O.

Y = 0.53 + 0.98X; X = pH values in CaCl_2_, Y = pH values in H_2_O. Where organic carbon values were reported as organic matter, it was converted using the following formula (Pluske et al., 2022):

Organic matter=1.72×Organic carbon

Soil textural classes were classified using USDA Soil Texture Calculator (USDA, 2022) based on percent sand, silt and clay for those studies which did not mention the textural class of soil. In addition, the locations of the experimental sites of the selected studies were also extracted to see the geographical distribution of microbial studies of neonicotinoids. To visualise different parameters of this systematic review, data were plotted in R software version 4.2.1.

## RESULTS

### Analysis of search strategy

**FIGURE 1:**
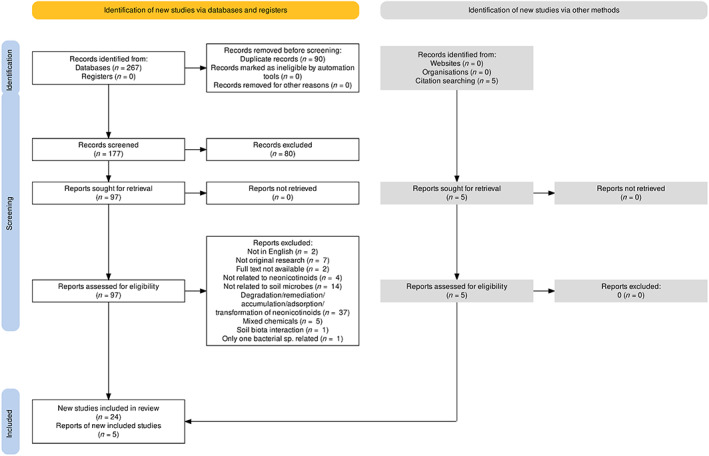
PRISMA (preferred reporting items for systematic review and meta‐analyses) flow diagram.

A total of 267 articles were found using keywords in SCOPUS, Web of Science and PubMed databases, and 5 articles were found by searching citations. After discarding repeated articles, 97 potential papers were identified for screening. Among these, 73 articles did not meet the inclusion requirements, hence they were excluded from the subsequent analyses. In general, this was due to experimentation on the degradation of neonicotinoids; or the absence of soil microbial experimentation. A sub‐set of 29 articles (Table 1) with 119 sampling points was then established following the methodology. The number of articles in each phase of the article selection process is depicted in the PRISMA flow diagram (Figure 1). Afterward, the sensitivity, precision and number needed to read (NNR) of the search strategy of this systematic review were calculated by the formula shown below (Lee et al., 2012; Lefebvre et al., 2022).

$$\text{Sensitivity}=\begin{array}{l} (\mathrm{no}.\;\text{of relevant studies identified}÷\text{total}\;\mathrm{no}.\\ \;\text{of relevant reports in the resource})\times 100 \end{array}$$

$$\text{Precision}=\begin{array}{l} (\mathrm{no}.\;\text{of relevant studies identified}÷\text{total}\;\mathrm{no}.\\ \;\text{of reports identified})\times 100 \end{array}$$

$$\text{Number needed to read}\;\left(\mathrm{NNR}\right)=1/\text{Precision}$$

**TABLE 1:** Summary of studies investigating neonicotinoids exposure to soil microbial population.

Author/study	Experiment	Exposure modality	Type of neonicotinoid	Country of study
Castillo Diaz et al., 2017	Field experiment	Spray application	Imidacloprid	Spain
Garg et al., 2021	Field experiment	Spray application	Imidacloprid	Not reported
Li et al., 2018	Field experiment	Seed treatment	Imidacloprid, Clothianidin	China
Mahapatra et al., 2017	Pot experiment	Spray application	Imidacloprid	India
Parizadeh et al., 2021	Field experiment	Seed treatment	Thiamethoxam	Canada
Wang et al., 2014	Laboratory experiment	Spray application	Imidacloprid, Acetamiprid	China
Wu et al., 2021	Laboratory experiment	Spray application	Thiamethoxam	China
Yu et al., 2020	Laboratory experiment	Spray application	Thiamethoxam, Dinotefuran	China
Zhang et al., 2018	Laboratory experiment	Spray application	Imidacloprid, Clothianidin, Thiacloprid	China
Cycoń & Piotrowska‐Seget, 2015a	Field experiment	Spray application	Imidacloprid	Poland
Cai et al., 2016b	Laboratory experiment	Spray application	Paichongding	China
Cycoń & Piotrowska‐Seget, 2015b	Field experiment	Spray application	Imidacloprid	Poland
Zhang et al., 2015	Laboratory experiment	Spray application	Imidacloprid	China
Astaykina et al., 2020	Laboratory experiment	Spray application	Imidacloprid	Russia
Cycoń et al., 2013	Field experiment	Spray application	Imidacloprid	Poland
Filimon et al., 2015	Field experiment	Spray application	Thiamethoxam	Romania
Ingram et al., 2005	Laboratory experiment	Spray application	Imidacloprid	Not reported
Streletskii et al., 2022	Laboratory experiment	Spray application	Imidacloprid	Not reported
Xiao et al., 2017	Laboratory experiment	Spray application	Imidacloprid	China
Zhang et al., 2021	Laboratory experiment	Spray application	Thiamethoxam	China
Yamaguchi et al., 2021	Laboratory experiment	Spray application	Dinotefuran	Japan
Cai et al., 2015	Laboratory experiment	Spray application	Paichongding	China
Cai et al., 2016a	Laboratory experiment	Spray application	Paichongding	China
Zhu et al., 2018	Laboratory experiment	Spray application	Paichongding	China
Deborah et al., 2013	Laboratory experiment	Spray application	Imidacloprid	India
Deborah & Madhuri, 2013	Laboratory experiment	Spray application	Imidacloprid	India
Singh & Singh, 2005a	Field experiment	Seed treatment	Imidacloprid	India
Singh & Singh, 2005b	Field experiment	Seed treatment	Imidacloprid	India
Singh & Singh, 2005c	Field experiment	Seed treatment	Imidacloprid	India

The sensitivity and precision of the search strategy were 28% and 11% respectively indicating low sensitivity and precision and as a result, reflecting low NNR (9.09).

### Experimental design and geographical distribution

Out of 29 selected studies, 4 were pot experiments, 8 were field experiments and the remaining 17 were laboratory experiments. Seven different neonicotinoids (dinotefuran, clothianidin, thiacloprid, thiamethoxam, acetamiprid, imidacloprid and paichongding) were tested, of which imidacloprid was used in 19 studies, thiamethoxam in 5 studies, paichongding in 4 studies, clothianidin in 2 studies, dinotefuran in 2 studies, acetamiprid in 1 study and thiacloprid was used in 1 study as a treatment for microbial studies. No study was found that used nitenpyram. Among all the studied neonicotinoids, paichongding is a cis‐nitromethylene type. About 83% of the selected studies (24 out of 29 articles) applied neonicotinoids as a spray and the remaining 17% of the studies (5 out of 29 articles) used neonicotinoids as a seed treatment.

**FIGURE 2:**
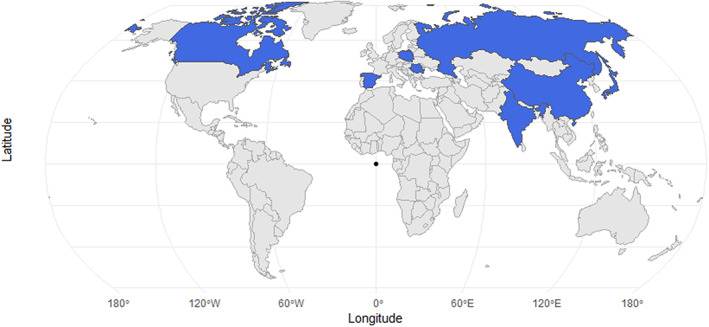
Geographical distribution of studies on neonicotinoid effects on soil microbial dynamics. Geographical reporting was at the country level.

In terms of the geographical location of neonicotinoid studies on soil microbes, the majority, 26 studies named the general locality of the sample sites of which, only 10 studies reported coordinate data and 3 studies did not specify study region or coordinates. We mapped all to acquire a global scale of relevant studies at the country level (Figure 2). The geographical distribution of studies of neonicotinoids on soil microbial communities reflected that all of the studies were conducted in the northern hemisphere with no work on neonicotinoids' impact on soil microbes found in South America, Africa and Oceania.

### Soil physicochemical features

**FIGURE 3:**
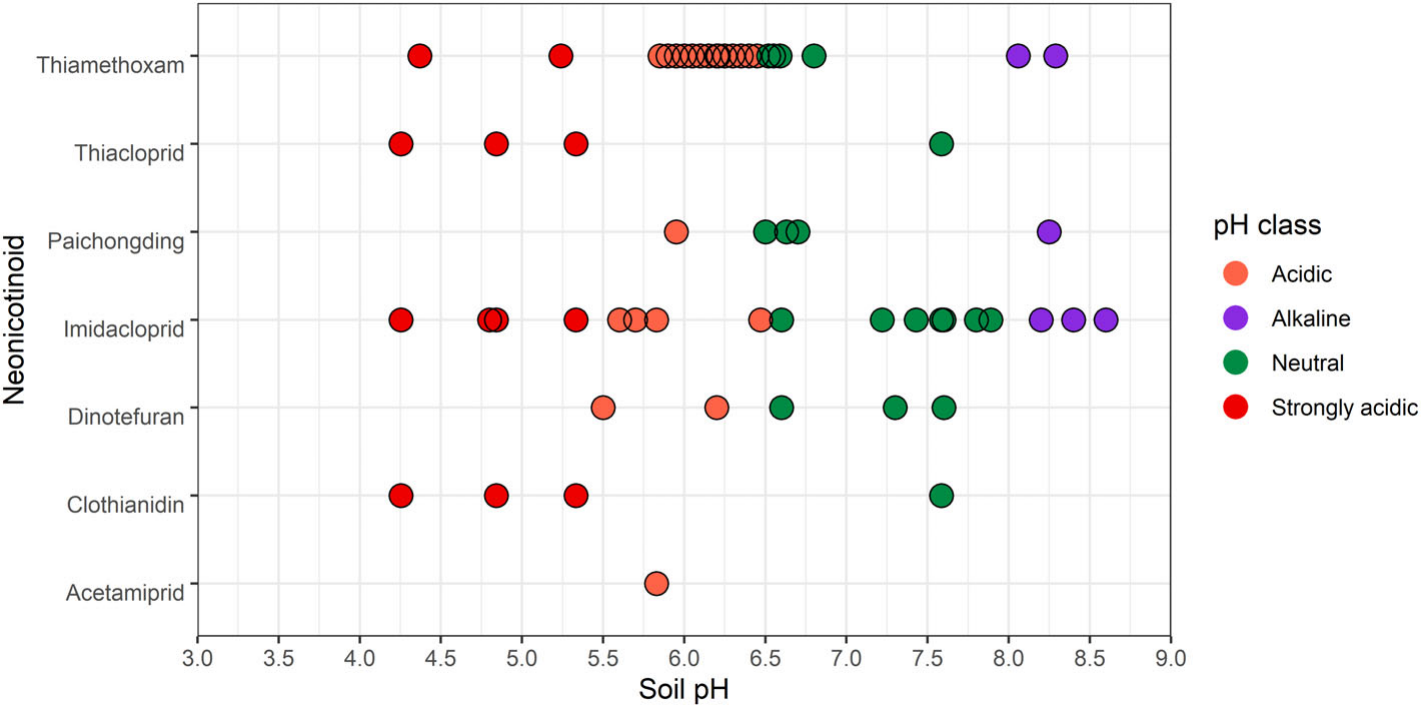
pH range of soils in selected studies. strongly acidic (pH <5.5); orange, acidic (pH 5.5–6.4); green, neutral (pH 6.5–7.9); and purple, alkaline (pH 8.0–9.2).

**FIGURE 4:**
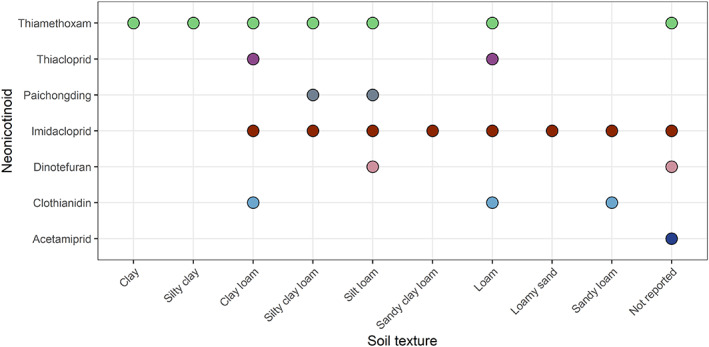
Soil textures in the selected studies on neonicotinoids. Each colour indicates a different type of neonicotinoid. Studies that did not mention the textural class were classified using the USDA Texture Calculator.

Soils used in all selected studies were collected from the field. For soil physicochemical factors, soil pH, organic carbon concentration, soil texture, clay, silt and sand concentrations, and soil depth data were extracted to visualise the extent of edaphic variation in the selected microbial studies. Soil pH was reported in 28 studies and 26 studies reported organic carbon concentration. 18 studies reported the clay, silt and sand concentration while 7 studies reported the textural classes of the soil without clay, silt and sand. Samples of soil in the selected studies were taken from the top 0.12–0.20 m of the rhizosphere. No pattern of changes in microbial populations with soil organic carbon was discussed in the selected articles. The experimented soils of the selected studies exhibited a wide range of pH (Figure 3). About 59% were acidic (pH 5.5–6.4), 23% were neutral (pH 6.5–7.9), 10% were strongly acidic (pH <5.5) and 6% were alkaline (pH 8.0–9.2). No strongly alkaline (pH > 9.2) soils were part of the selected data set. Likewise, a wide range of textural classes was present in the selected papers (Figure 4). About 37% of the studied samples were clay while the least was loamy sand (3%).

### Microbial parameters

**FIGURE 5:**
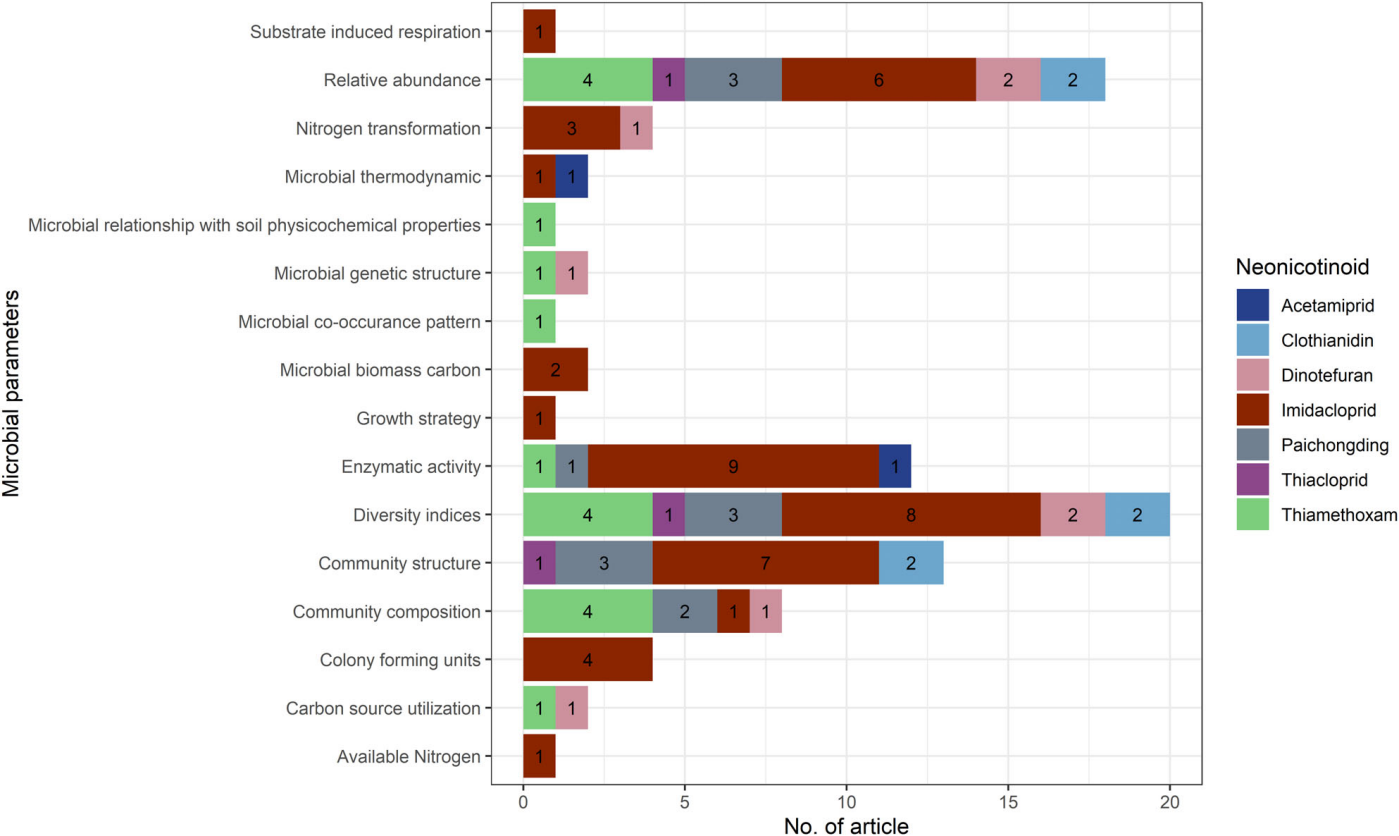
Microbial parameters of selected studies after exposure to neonicotinoids. Each colour indicates a different type of neonicotinoid and each number within horizontal bars is the number of articles for an individual neonicotinoid.

We observed that 16 different microbial parameters were assessed by the various authors when determining the impacts of neonicotinoids on soil microbial populations. Diversity indices were reported most frequently (18 studies) followed by relative abundance (14 studies), enzymatic activity (11 studies), community structure (10 studies) and community composition (6 studies) (Figure 5). Diversity indices included abundance‐based coverage estimators (ACE), shannon index, simpson index and chao1. Relative abundance was measured as the percent composition of a microorganism of a particular kind relative to the total number of microorganisms in the area. In terms of enzymatic activity, dehydrogenase, urease, β‐glycosidase, fluorescein diacetate hydrolase, acid phosphatase, phosphomonoesterase, protease, catalase, phosphatase, amylase and arginine deaminase activity were assessed in the selected studies. Community structure was monitored by measuring the genetic variability and community composition was measured by analysing which species were present and the abundance of their members.

### Neonicotinoid studies that reported no adverse effect on the soil microbial population

Out of 29 selected studies, only five (17%) found no or very limited significant adverse effects on the relative abundance and diversity of microbial populations, some of which are responsible for the biodegradation of neonicotinoids from the neonicotinoids in question (imidacloprid, clothianidin and thiamethoxam) (Garg et al., 2021; Li et al., 2018; Parizadeh et al., 2021; Singh & Singh, 2005a; Zhang et al., 2015). PLFA, PCR‐DGGE and CLPP approaches used by Cycoń et al. (2013) revealed that imidacloprid applied at field rate had a negligible impact on the community structure and biodiversity of native soil microorganisms. Wu et al. (2021) reported that thiamethoxam application had no significant effects on some soil metabolic processes such as the carbon metabolism (tricarboxylic acid cycle, pentose phosphate and glycolysis), nitrogen cycle and sulphur metabolism. Some studies reported that imidacloprid stimulates plant available nitrogen, nitrification, ammonification and nitrogen fixation processes in soil (Astaykina et al., 2020; Cycoń & Piotrowska‐Seget, 2015a; Singh & Singh, 2005c; Zhang et al., 2018), while dinotefuran promotes ammonia oxidation (Yamaguchi et al., 2021). In terms of enzymatic activity, some studies found that imidacloprid had no adverse effect on microbial phosphatase, protease and urease activity in soil (Deborah & Madhuri, 2013; Ingram et al., 2005). Some studies reported that amylase, dehydrogenase, phosphomonoesterase and arginine deaminase activity was increased at field rate application of imidacloprid and acetamiprid (Singh & Singh, 2005c, b; Deborah et al., 2013; Wang et al., 2014). Cai et al. (2016b) reported that dehydrogenase, protease and catalase activities were all stimulated differently by paichongding. In low‐saline soils, the soil bacterial diversity marginally decreased with increasing imidacloprid concentration, however, it might somewhat boost the soil bacterial diversity in moderately saline soils (Zhang et al., 2015).

### Neonicotinoid exposure studies that reported adverse effects on soil microbial populations

Thirteen studies (45%) discussed the adverse effect of neonicotinoids (clothianidin, dinotefuran, imidacloprid, paichongding, thiacloprid and thiamethoxam) on soil microbial relative abundance, diversity, activity, community composition and community structure. Imidacloprid changed the relative abundance of some eukaryotic genera named *Apiotrichum*, *Gamsia*, *Humicola*, *Kitasatospora*, *Solicoccozyma* and prokaryotic genera named *Sphingomonas*, *Streptomyces* and *Terrabacter* (Streletskii et al., 2022). In comparison to bacteria, low relative abundances of the archaea genera *Crenarchaeota* and *Euryarchaeota* were found in loam and clay loam soil treated with imidacloprid, thiacloprid and clothianidin (Zhang et al., 2018). Parizadeh et al. (2021) reported that the relative abundance of various potentially beneficial soil bacteria, notably the rhizobacteria that encourage plant development, decreased after neonicotinoid seed treatment. Cai et al. (2016b) found that the actinomycic population and the cultivable fungal population significantly decreased at the beginning and after 60 days of the experiment respectively. In the study of Mahapatra et al. (2017), imidacloprid was applied at 4 different doses (recommended dose, double, 5 times and 10 times the recommended dose) and the results showed that imidacloprid treatment resulted in a decrease in the overall soil microbial biomass carbon and higher concentration of imidacloprid decreased bacterial, actinomycetes and fungal population. The reduction in bacterial and fungal population was 3 and 6.3% less, respectively, in the 10 times of the recommended dose treatment compared to control. Imidacloprid resulted in a reduction in the total of selected PLFA (phospholipid fatty acid) concentrations when compared to the controls, demonstrating an inhibitory action on soil bacteria and fungus (Xiao et al., 2017). Cycoń et al. (2013) reported that imidacloprid applied at a higher rate significantly altered the composition of the microbial community and reduced the biomass of the total, bacterial and fungal PLFAs as well as lower their metabolic activity. Imidacloprid sensitivity had been found in the Ammonia‐Oxidizing Archaeal community in some nitrifying and N_2_‐fixing bacteria, potentially posing a threat to nitrogen cycles in soil (Cycoń & Piotrowska‐Seget, 2015a, 2015b). Regardless of soil type, nitrite oxidation and *Methylotenera* spp. richness was suppressed when dinotefuran was used in high concentrations (Yamaguchi et al., 2021). In anaerobic soil, paichongding had been found to inhibit bacterial diversity as well as growth (Cai et al., 2015) and the soil bacterial community composition varied at both phylum and genus levels (Cai et al., 2015; Cai et al., 2016a). Paichongding also caused significant differences in eukaryal population before and after application and it could cause some phyla and genera to grow or shrink in anaerobic soils (Zhu et al., 2018). Five studies (17%) reported that acetamiprid, imidacloprid, paichongding and thiamethoxam inhibited soil enzymatic activities (cellulase, urease, dehydrogenase, catalase, phosphatase and phosphomonoesterase) ultimately hampering biogeochemical function of soil such as ammonification, nitrification and denitrification (Cai et al., 2016a; Castillo Diaz et al., 2017; Deborah et al., 2013; Filimon et al., 2015; Wang et al., 2014). Out of 24 studies, only one study (3%) reported that thiamethoxam application reduced soil bacterial co‐occurrence patterns, which also depend on soil pH and cation exchange capacity (Zhang et al., 2021).

## DISCUSSION

Despite the fact that a lot of time and effort has gone into researching and debating neonicotinoids' environmental effects, rigorous assessments of their soil ecotoxicology are rare. To our knowledge, this is the first systematic review to explore soil microbial health of exposure to neonicotinoids in the peer‐reviewed literature. To address the possible changes in soil microbial communities after exposure to neonicotinoids, this systematic review was performed using the PRISMA guidelines, an approach commonly used in agro‐environmental research (Koutsos et al., 2019; Moher et al., 2010; Vásquez‐Dean et al., 2020).

Sensitivity (28%), precision (11%) and number of needed to read (9) metrics were calculated to evaluate the search strategy. The goal of the systematic review search strategy was to be as broad as feasible in order to include as much relevant research as possible in the review. A search strategy that strikes an equilibrium between high sensitivity and high precision is known as an optimum search (Petitti, 2000). Designing a search strategy always starts with a study of the key ideas and the keywords that will be used to describe each notion (Salvador‐Oliván et al., 2019). Higher sensitivity eventually reduces the precision percentage and ultimately retrieves non‐relevant studies in return (Dieste & Griman, 2007; Lefebvre et al., 2022). For instance, lower sensitivity and precision indicated that the search strategy extracted more relevant studies with higher accuracy but the reference standard only comprised a small number of papers, which might impair the accuracy of the performance evaluation (Dieste et al., 2009; Li et al., 2019). The NNR (number of needed to read) of the search strategy reflects that nine articles were read to find a relevant study to be included in this systematic review indicating substantially lower screening time.

An insight into the possible changes in soil microbial activity after applying neonicotinoid insecticides was provided by this approach, and through the analysis of the selected articles knowledge gaps were identified. A scarcity of research articles related to the topic was a major limitation of this review. A total of 272 articles were found using the keywords in PubMed SCOPUS and Web of Science databases, and citation searching, while changing only the keyword ‘neonicotinoid’ to ‘glyphosate’ resulted in 1115 articles. From the view of the geographical distribution of 29 studies identified through this systematic approach, there was no study in the southern hemisphere of the world. According to Bass et al. (2015) Latin America, Asia and North America account for 75% of the world's neonicotinoid usage, with Europe making about 11% of overall sales. But from geographical distribution, it had been reflected that no microbial studies on neonicotinoid were conducted in Latin America. On February 28, 2018, the European Food Safety Authority (EFSA) website posted the conclusions of its risk assessment in relation to affecting bees and other environmental threats for the active ingredients imidacloprid, thiamethoxam and clothianidin, therefore, the recommendations to entirely outlaw the outdoor usage of these three active ingredients were maintained by the Commission services and endorsed by a major of Member States in the Regulatory Committee on April 27, 2018 (EFSA, 2018). On the other hand, a majority portion of the world still does not have sufficient evidence to assess the impact of neonicotinoids on soil biology and ecology. Different regions of the world have different climates‐ thus resulting in distinct soil development patterns. These facts also highlighted the need for soil microbiological research on neonicotinoids in order to have a comprehensive understanding of the physicochemical and microbiological features of soil after exposure to neonicotinoids across the world.

The reported data in the selected studies often differed among themselves. Most were carried out in the laboratory, with few occurring in the field and all had variable exposure modalities, thus, true meta‐analysis was difficult to perform. On the other hand, studies on all commercially available neonicotinoids were scarcer which indicates more diverse group of neonicotinoids should be tested on soil microbial communities. For a better understanding of neonicotinoids‐soil microorganism complexities, a more seamless transition from laboratory to field application is required because soils provide a wide range of crucial ecosystems that have components that might impact pesticide effects (de Santo et al., 2019; Kördel & Römbke, 2001; Parnell et al., 2016). Pesticide toxicity on soil microbes relies on soil characteristics, pesticides’ physical and chemical behaviour, concentrations and mode of application (Kalia & Gosal, 2011). Foliar application and seed treatment of pesticides react differently in soil. According to Moorman (1989) foliar‐sprayed pesticides concentrated on the soil surface initially but seed treatment created higher concentration of pesticides near the seed. In this systematic review, fewer numbers of microbial studies were found where the mode of application was seed treatment despite that globally, neonicotinoids are widely applied as seed treatment (Mourtzinis et al., 2019).

Important physicochemical drivers of soil microbial productivity were poorly controlled in the selected studies. Only one study reported the relation between microbial co‐occurrence pattern and soil physicochemical properties and found that edaphic variables significantly influenced the response of soil bacterial networks to neonicotinoids (Zhang et al., 2021). Soil physicochemical properties are also strongly related to soil microbial activity and diversity (Buyer et al., 2010; Fierer et al., 2012) as well as the fate and behaviour of neonicotinoids in soil (Aseperi et al., 2020).

We also found that research on the effects of neonicotinoids on soil microorganisms has produced conflicting results. Some studies did not find any adverse effects of neonicotinoids on microorganisms (Garg et al., 2021; Li et al., 2018; Parizadeh et al., 2021; Singh & Singh, 2005a; Zhang et al., 2015), while some found significant changes in the microbial community in terms of relative abundance (Garg et al., 2021; Parizadeh et al., 2021; Streletskii et al., 2022; Wu et al., 2021; Zhang et al., 2018), diversity and structure (Cai et al., 2015; Wu et al., 2021; Yamaguchi et al., 2021; Yu et al., 2020; Zhang et al., 2015; Zhang et al., 2018; Zhang et al., 2021) and enzymatic activity (Cai et al., 2016a; Castillo Diaz et al., 2017; Deborah et al., 2013; Filimon et al., 2015; Wang et al., 2014). Such contradictory results may be because some authors investigated only eukaryotes, some only prokaryotes, and others a combination of the two groups. In our systematic review, we found that 13 studies had been conducted on prokaryotes, one on eukaryotes and five on both prokaryotes and eukaryotes. Recent studies have also challenged the notion that neonicotinoids primarily impact eukaryotic soil microbes (Wang et al., 2023) because they share some cellular features with insects (Lemelin & Fine, 2013) such as eukaryotic cell structure (Cooper, 2000), chitin in cell wall/exoskeleton (Numata & Kaplan, 2011) and cytoplasmic streaming (Goldstein & van de Meent, 2015).

Although neonicotinoids as group of target invertebrates (i.e., insects) and most soil microbes do not possess the specific target proteins for neonicotinoids, they can still be indirectly affected by these insecticides. Any pesticide can interact with enzymes in soil microorganisms, leading to unintended effects on their physiology and metabolism (Das et al., 2005; Floch et al., 2011) that had also been observed in neonicotinoids (Cycoń & Piotrowska‐Seget, 2015a). When any change or disturbance occurs in the surrounding environment of microbial communities, they modify their pattern of habitat by increasing their abundance, or become more robust to the disturbance over time (Fierer et al., 2010; Redford & Fierer, 2009) but, concurrently, there may have been a commensurate decline in functional microbial diversity (Tripathi et al., 2020). Long‐term inconsistent and excessive pesticides usage disrupt the stability of microbial population (Lupwayi et al., 2009) hence making biogeochemical nutrient cyclings like nitrogen cycle and organic matter mineralisation sensitive to pesticide (Jat et al., 2021; Sim et al., 2022; Xu et al., 2001). On the other hand, the soil nutrient status was poorly addressed within the selected studies on neonicotinoids, and the findings may well reflect changes in soil nutrient conditions rather than their impacts on soil microbial populations.

Concerns have also been raised about the adverse effects of neonicotinoids on non‐target soil fauna. Neonicotinoid seed treatment reduced earthworm growth, activity, behaviour and burrowing capacity (Capowiez et al., 2005; Capowiez et al., 2006; Ge et al., 2018) and soil treated with neonicotinoids at the concentration of 1 ppm or higher can pose mortality risk to earthworms (Pisa et al., 2015). Neonicotinoid exposure in soil resulted in a large increase in adult DNA damage as well as a significant reduction in earthworm reproduction that occurred concurrently with neonicotinoid bioaccumulation (Chevillot et al., 2017). Yu et al. (2021) reported that the relationships between Collembola, fungi and bacteria that regulate the mineralisation of maize and soil organic carbon are changed by dinotefuran. Clothianidin had been found to reduce the population of earthworms, springtails (de Lima e Silva et al., 2020) and oribatid mites (Ritchie et al., 2019).

Wang et al. (2023) investigated the responses of eukaryotic soil protozoa to imidacloprid exposure and found evidence of altered key differentially expressed genes involved in cell division, cytochrome P450, morphogenesis and phagocytosis pathways.

In summary, our findings suggest that although the effects of neonicotinoids on soil microbial life, in general, remain unclear, some detrimental effects on soil microbial and enzymatic activity were reported by a number of authors.

In this systematic review, we identified the following research gaps:

- Most research on neonicotinoids and soil microorganisms had been conducted in laboratory settings or controlled environments with very few field studies. Consequently, more field‐scale studies are needed to understand how neonicotinoids influence soil microbial dynamics under real‐world agricultural conditions.
- The existing literature is focused on foliar spray application while the effects of seed treatments remain understudied. Thus, there is a need for more spray versus seed treatment comparison experiments.
- The relationship between specific physicochemical properties of soil and the observed changes in microbial habitat caused by neonicotinoids remains relatively unclear. Determining the key soil properties that are most influenced by neonicotinoid exposure and how they mediate microbial responses is crucial for targeted conservation and management strategies.
- A knowledge gap exists with respect to the impact of neonicotinoids on nutrient availability, and consequently, modify microbial community dynamics.
- While the primary neonicotinoid compounds had been extensively studied, there is a lack of comprehensive research on the formation and fate of their metabolites in various environmental settings. Understanding the transformation pathways and persistence of these metabolites is essential for assessing their potential impacts on ecosystems and non‐target organisms.
- The lack of comprehensive studies on the effects of neonicotinoids on soil eukaryotic microbes hinders our ability to fully assess the broader impacts of these chemicals on soil biodiversity and ecosystem services. Furthermore, it may limit the development of targeted and sustainable pest management strategies that consider the interactions between neonicotinoids and diverse soil microbial communities. To gain a comprehensive understanding of the impacts of neonicotinoids on eukaryotic microbes, more targeted studies are warranted to investigate the mechanisms of toxicity, the potential for resistance or adaptation and the ecological consequences of such effects on soil microbial communities.

## CONCLUSION

The current information on the microbial communities in relation to the exposure of neonicotinoids was compiled and visualised by this systematic review through an integrative approach. Due to diverse methodologies with different research outputs, a comprehensive meta‐analysis was difficult to perform. The severity of neonicotinoid toxicity on soil microbes was determined by the dose and duration of exposure. Imidacloprid was the mostly used (66%) neonicotinoid class in soil microbial studies and the soil microbial population was sensitive to it as exposure to unfavourable physiological states could have detrimental effects on their survival and growth, leading to a decrease in their abundance eventually affecting metabolic processes. Laboratory studies dominated over field experiments for the studies of microbial parameters in neonicotinoid‐exposed soil. Even though heterogeneity was high, 45% of the selected studies found significant negative impacts on soil microbial community structure, composition, functioning, diversity, enzymatic activity and nitrogen transformation after neonicotinoid exposure. The scarcity of studies highlighted the need for further research on this topic.

## AUTHOR CONTRIBUTIONS

**Sharmin Akter:** Conceptualization (equal); methodology (lead); writing – original draft (lead). **Nilantha R. Hulugalle:** Supervision (supporting); writing – review and editing (lead). **Julia Jasonsmith:** Conceptualization (equal); supervision (supporting); writing – review and editing (supporting). **Craig L. Strong:** Conceptualization (equal); supervision (lead); writing – review and editing (supporting).

## CONFLICT OF INTEREST STATEMENT

The authors declare no conflict of interest.

## Data Availability

The data that support the findings of this study are available from the corresponding author upon reasonable request.

## Footnotes

^1^
PRISMA stands for Preferred Reporting Items for Systematic Reviews and Meta‐Analyses. It is an evidence‐based minimum set of items for reporting in systematic reviews and meta‐analyses which consists of a 27‐item checklist and a 4‐phase flow diagram. PRISMA primarily focuses on the reporting of reviews evaluating the effects of interventions, but can also be used as a basis for reporting systematic reviews with objectives other than evaluating interventions (e.g., evaluating aetiology, prevalence, diagnosis or prognosis).
